# Unmet need of long-acting and permanent family planning methods among women in the reproductive age group in shashemene town, Oromia region, Ethiopia: a cross sectional study

**DOI:** 10.1186/s12905-015-0209-y

**Published:** 2015-07-15

**Authors:** Kuma Mota, Surender Reddy, Biniam Getachew

**Affiliations:** Marie Stopes International Ethiopia, P. O. Box: 329, Hawassa, Ethiopia; Wollo University Ethiopia, P. O. Box: 1145, Dessie, Ethiopia; Ethiopian Society of Obstetricians and Gynecologists, P. O. Box: 27198/1000, Addis Ababa, Ethiopia

**Keywords:** Long Acting and Permanent Methods (LAPMs), unmet need of LAPMs & Family planning

## Abstract

**Background:**

Many countries continue to have high fertility rates and most of the predicted increase in the world’s population until 2100 comes from these countries. Among family planning methods, Long Acting and Permanent Methods are convenient for users and effectively prevent pregnancy. The objective of this study was to assess factors associated with unmet need of Long Acting and Permanent Methods of contraception among women in the reproductive age group (15-49) using contraception in health facilities of Shashemene town, Oromia Region, Ethiopia.

**Methods:**

Facility based cross sectional quantitative study and stratified sampling technique was used. Total of 382 females in reproductive age group were enrolled in the study in January 2012. Pretested, structured and close-ended questionnaire was used to interview study participants. Collected data was entered by using EPI Info 17 and analyzed by SPSS version 20 statistical software.

**Results:**

Utilization of Long Acting and Permanent Methods (LAPMs) of contraception in Shashemene town was found to be 28.4 % (104/366). From study participants, 71.6 % (262/366) used short acting methods and from these current short acting users, 127(41.5 %) desired to use LAPMs and unmet need of LAPMs was 122(33.3 %). Factors significantly associated with unmet need of LAMPs of contraception were: Education of women (< secondary level) AOR [3.8, 95 % CI: 2.9, 7.6; P < 0.001]; lack of discussion between partners AOR [2.9, 95 % CI: 1.8, 9.6; P = 0.01]; lack of proper counseling for women AOR [5.3, 95 % CI: 1.7, 11.2; P = 0.04]; and women’s occupation as a housewife AOR [4.7, 95 % CI: 3.1, 11.3; P = 0.02].

**Conclusion:**

Unmet need of LAPMs of contraception in health facilities in Shashemene town was high. Women education, partner discussion and proper client counseling were found the main factors associated women LAPMs utilization.

## Background

Four contraceptive methods are categorized as long-acting and permanent (LAPMs): intrauterine contraceptive devices (IUCDs), implants, female sterilization, and vasectomy. IUCDs and implants are long acting temporary methods; when removed, return to fertility is prompt. Copper-containing IUCDs, the ones generally available in African Ministry of Health (MoH) family planning programs, is effective for at least 12 years, although it is labeled for 10 years. Implants, depending on the type, last for up to three to seven years. Female sterilization or tubal ligation and vasectomy are permanent methods [1].

Unmet need for family planning is defined as percentage of all fecund women who are married or living in union and thus presumed to be sexually active but are not using any method of contraception, either do not want to have more children or want to postpone their next birth for at least two more years or do not know when or if they want another child. The concept of unmet need points to the gap between women’s reproductive intentions and their contraceptive behavior [2, 3].

In developing countries, 20 to 30 % of women who use oral contraceptives or injectable stop within two years of starting because of side effects or other health concerns. Many of these women could benefit from switching to LAPMs [4]. Many countries continue to have high fertility rates and most of the predicted increase in the world’s population until 2100 comes from these high-fertility countries [5]. Ethiopia’s Reproductive Health Strategy (2006–2015) identifies priority areas to improve the reproductive health status of this country and fertility & family planning is one of the six strategies [6].

Ethiopia is a second populous country in Africa with a total population of 90,076,000 [7, 8]. The prevalence rates of contraceptives (CPRs) were 8.1 %, 14.7 % and 29 % in 2000, 2005 and 2011 respectively, while the unmet needs for family planning during the same periods were reported as 36 %, 34 % and 29 % respectively [9, 10]. Even though, LAPMs are effective and convenient method of pregnancy prevention for users, the prevalence of LAPMs remains relatively small with only accounts of 3.9 % (EDHS 2011) and sometimes missing component of national reproductive health and family planning programs [10, 11].

Evidence from studies in South East of and North West of Ethiopia shows unmet need for LAPMs was 9.4 % and 16.4 %, respectively [12, 13]. Data from Ethiopian demographic and health survey shows unmet need for married women to be 25 %, met need 29 % and the total demand 53.9 % and the prevalence of LAPMs of FP services in whole Ethiopia is about 3.9 %. But in Oromia, unmet need for modern FP 29.9 %, met need for modern FP 26.2 % and the total demand for modern FP 56.1 % and the prevalence of LAPMs of family planning services in Oromia Region is about 6 % [9]. According to Shashemene town health office report of 2011, the FP service coverage was 92 % but no clear data about the prevalence of LAPMs [14]. Therefore, this study intended to assess the extent of unmet need for LAPMs and contribute factors associated with unmet need of LAPMs of FP services in Shashemene town.

## Methods

The study was conducted in Shashemene town of West Arsi zone, Oromia National Regional state, which is located around 240 Km from Addis Ababa (capital city) to the south of Ethiopia. Shashemene town has a total population of 140,717 of which 70,339 are women and women of childbearing age group expected to be 15,566 [8]. At the time of the study, there were 16 health institutions providing modern family planning to the community: 13 public health institution, 2 NGO clinics and one private medium clinic. The study area is selected due to the non exixtence of clear data on met and unmet need of LAPMs in the town.

Facility based cross sectional quantitative study was conducted in January and February, 2012, to determine the extent of unmet need of LAPMs of family planning services among women in the reproductive age group using modern family planning in the health facilities

The sample size was calculated for the unmet need of LAPMs (using single proportion formula) and factors associated with unmet need of LAPMs (double proportion formula). And the sample size of extent of unmet need of LAPMs was higher than factors associated with unmet need of LAPMs, we use sample size of extent of unmet need of LAPMs: n = 382.

### Assumptions

Desired precision/margin of error (**d**) = 3.75 %, the prevalence of unmet needs of LAPMs in Oromia Region (**p**) was about 15 % and **CI** = 95 %, which means **α** set at 0.05 and **Ζ α/2** = 1.96 (value of **Ζ at α** 0.05 or critical value for normal distribution at 95 % CI) by calculating with Epi Info 7 statcalc. Hence, the calculated sample size was found to be **348 + 10 % (**non-response rate) **= 382.**

In Shashemene town there were 8 health facilities (by excluding 8 health posts because of non- fixed facility) providing modern FP services. A stratified sampling was used by dividing 8 health facilities in to strata: 4 health facilities with high client flow and 4 health facilities with low client flow. A separate sample was taken independently from each stratum by using simple random sampling. Thus, 3 health facilities out of 4 health facilities with high client flow and 3 health facilities out of 4 health facilities with low client flow were selected. Record review indicating how many clients received modern family planning services in the last 4 completed quarters (12 months) was made in each stratum, and then proportional allocation was used for each stratum to select study subjects.

Data was collected continuously by exit interview from study subjects who came for modern family planning within the study period in 6 selected health facilities until the required sample size was obtained according to their proportional size. The questionnaire was adapted from other studies developed for similar purposes [15, 16] and some adjustment was made to suit the local condition and study objective.

The study data were collected, checked, edited for consistency, processed and analyzed generally by means of a package computer program: statistical soft-wares: EPI Info 7 for data entry format designing, data entry, data cleaning and data processing/management; and SPSS 20 used for data analysis. The cut point that was used for statistical significance was the P- value 0.05.

### Ethical considerations

Ethical clearance was obtained from Hawassa University and Addis Continental Institution of Public Health. Official permission was collected from Shashemene town Health Department, and both written informed consent and verbal was obtained from individual participants. All study participants in the survey were told about their participation was voluntary and confidential.

### Operational definition of key words in unmet need of LAPMs is given below:

- Unmet need for LAPMs of Contraceptive: - A condition of wanting to postpone or avoid pregnancy but not using any of the LAPMs of contraceptive or using short acting contraceptive but do not wants to use the method that they were using.
- Short acting contraceptive: - Pills, injectable hormones and condoms.
- Long acting contraceptive: - Intra-Uterine Contraceptive Device (IUCD) and Implants.
- Permanent methods of contraceptive: - Tubal ligation and vasectomy. However, vasectomy not belongs to this operational definition since no male participated.
- Unmet need for limiting: - When women who do not want any more children, but not using any of LAPMs of contraceptive.
- Unmet need for spacing:- When a woman who does not want to have pregnancy soon after delivery or want to space for two years but not using any of LAPMs of contraceptive.

## Results

### Socio-demographic characteristics of the respondents

A total of 382 study subjects were participated in the survey. The mean age of the respondents was 29.6 ± 7.8 S.D and the median age was 28 years ranging from 15-46 years. The highest proportion of the respondents 301/382 (78.8 %) were in the age group 15–34 years. Almost all 366 (95.8 %) of the respondents were married and living together. Majority of the respondents 376 (98.4 %) were residing in urban areas. About 91 (23.8 %) of respondents had no education at all; while the remaining 291 (76.2 %) attended school ranging from primary to secondary and above. The predominant ethnic group of the respondents was Oromo 49.2 % (188) followed by Amhara 42.4 % (162). The religion of most respondents was Orthodox, 39.8 % (152), followed by Muslim 32.5 % (124). The occupation of respondents was 33 % (126) house wife followed by government employee 19.7 % (75). The monthly income of the majority of the respondents 55.2 % (211) was 1,500-3,499 ETB followed by 31.9 % (122) was <1500 ETB (Table 1).

**Table 1:** Socio-demographic characteristics among women in the reproductive age group (15-49) using contraception in health facilities in Shashemene town, West Arsi Zone, Oromia Region, 2012 (n = 382)

Characteristics	Number	Percent
Age Group (years)		
<25	154	40.3
25-34	147	38.5
> = 35	81	21.2
Mean ± SD for women15-49 years. =29.6 ± 7.8 and Median age for women15-49 years. =28 years.		
Marital status		
Single	9	2.4
Married	366	95.8
Separated	6	1.6
Divorced	1	0.26
Residence		
Town	376	98.4
Rural	6	1.6
Education		
Illiterate	91	23.8
Primary (1–8)	110	28.8
Secondary (9-10) and above	181	47.4
Ethnicity		
Amhara	162	42.4
Oromo	188	49.2
Others^a^	32	8.4
Religion		
Orthodox	152	39.8
Muslim	124	32.5
Protestant	106	27.7
Occupation		
House wife	126	33
Government employee	75	19.7
Daily laborer	23	6
Student	52	13.6
Merchant	96	25.1
Others^b^	10	2.6
Income		
<1500	122	31.9
1500-3499	211	55.2
> = 3500	49	12

^a^ = Wolaita, Kambata, Hadiya; ^b^ = Tella (local alcohol drink) Sellers/self-employee

The mean age of women at marriage and first delivery was 18.02 ± 2.4 SD and 19.6 ± 2.6 SD years respectively. The average number of pregnancies was 2.57 ± 0.8 SD, out of which, 59.9 % (217/362) experienced three and above pregnancy. Two hundred one (64.8 %) of the women aged 15-49 years responded that they want to have any more children in the future, of which one hundred thirty-one (65.2 %) seek 1-3 children in their life time. Most women one hundred seventy-two (85.6 %) want to have any more children after two years and above. Forty five (12.3 %) of the women aged 15-49 years had abortion, of which 10 (22.2 %) experienced more than one induced abortion in their life time. The average number of children in the household was 1.35 (SD = 0.7) (Table 2).

**Table 2:** Reproductive History among women in the reproductive age group (15-49) using contraception in health facilities in Shashemene town, West Arsi Zone, Oromia Region, 2012

Characteristics	Number	Percent
Age at marriage (n = 366)		
<18	190	51.9
≥18	176	48.1
Age at delivery (n = 317)		
<18	44	13.9
≥18	273	86.1
Number of pregnancy (n = 362)		
One/two	145	40.1
Three/four	112	30.9
Five or more	105	29.0
Number of living children (n = 310)		
One/two	133	42.9
Three/four	95	30.6
Four or more	82	26.5
Do you want to have any more children? (n = 310)		
Yes	201	64.8
No	109	35.2
Ideal number of children you want (n = 201)		
1-3	131	65.2
4 or more	45	22.4
Depend on God	2	1.0
Depend on husband	23	11.4
When you want to have any more children? (n = 201)		
Less than two years	29	14.4
Two years and above	172	85.6
Number of abortion (n = 45)		
One	35	77.8
Two and above	10	22.2

### Unmet need of LAPMs

**Fig. 1:**
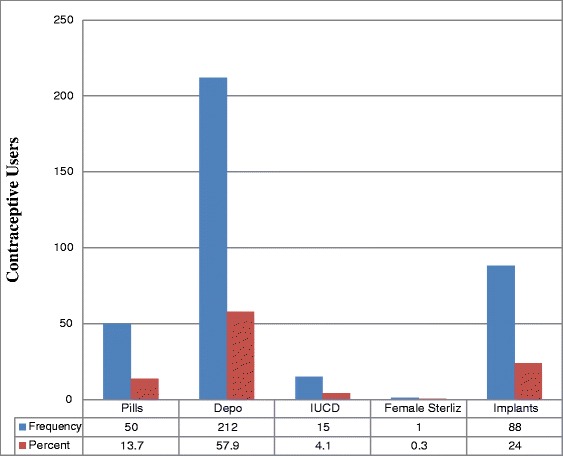
Contraceptive Methods Currently in Use among Women in Reproductive Age Group (15-49) in Health Facilities in Shashemene Town, West Arsi Zone, Oromia Region, 2012

Further analysis carried out on the married respondents of 366 women. The current utilization rate of LAPMs of contraceptives in the town was 104 (28.4 %). Of these 88 (24 %) were using Implants, 15 (4.1 %) IUCD and 1 woman (0.3 %) were using Tubal ligation (Fig. 1). Two hundred sixty two (71.6 %) of the respondents were using short acting contraceptives mainly Depo-Provera 212 (57.9 %); and pills 50 (13.7 %). Out of these short acting contraceptive users 135 (51.5 %) were preferred to use the method and 127 (41.5 %) of these current short acting contraceptive users did not want to use the method that they were using (Table 4).

**Fig. 2:**
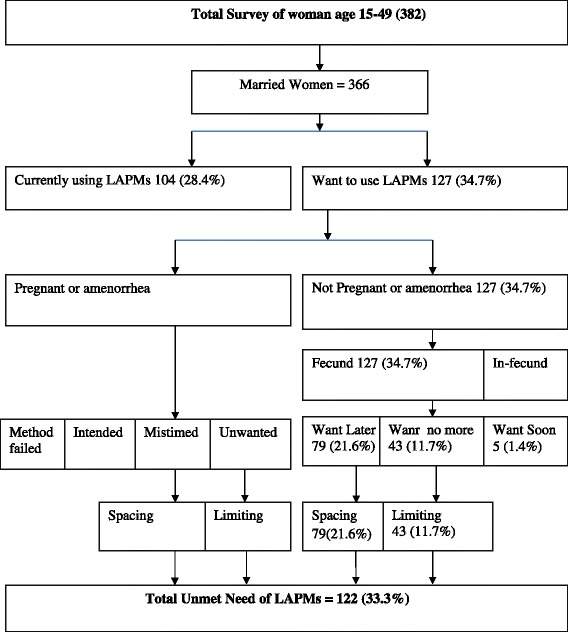
Unmet Need of LAPMs among Women in The Reproductive Age Group (15-49) Using Contraception in Health Facilities in Shashemene Town, West Arsi Zone, Oromia Region, 2012 (Westoff Model)

The total unmet need of LAPMs of contraceptives in health facilities in the town was found to be 122 (33.3 %) of which seventy-nine (21.6 %) for spacing and forty-three (11.7 %) for limiting (Fig. 2).

### Associated factors and unmet need of LAPMs

Most of the respondents (85 %) heard about LAPMs of contraceptives at least once. From which 362 (98.9 %) heard about implants as LAPMs of contraceptive and the least ever heard LAPMs of contraception was Tubal-ligation 220 (60.1 %). Less than one-fourth 82 (22.4 %) of the respondents were counseled about LAPMs of contraception by health professionals. Nearly all 355 (97 %) reported, knew the source of LAPMs of contraceptives were found in health facilities in the town. Three hundred twenty four (88.6 %) have heard of LAPMs from Hospital sources. Media (radio and television) was the major sources of information 322 (88 %). In the past few months only 146 (39.9 %) respondents discussed about LAPMs once/twice and 41 (11.2 %) respondents discussed about LAPMs more often with their husband/partner. One hundred seventy nine (48.9 %) of the respondents had never discussed about LAPMs of contraceptives with their husband/partner. One hundred ninety eight (54.1 %) of the respondents responded that their husband/partner opposes using LAPMs, while 192 (41.8 %) of the respondents responded their husband/partner approves using LAPMs. The decision of using LAPMs of contraceptive was made by both partners together for 235 (64.2 %) of the respondents (Table 3).

**Table 3:** Percentage of ever heard, LAPMs counseling, Knowledge of source, discussion with husband and source of information of LAPMs among women in the reproductive age group (15-49) in Shashemene town, West Arsi Zone, Oromia Region, 2012 (n = 366)

Characteristics	Number	Percent
Ever heard at least LAPMs		
IUCD	355	97.0
Implants	362	98.9
Tubal-ligation	220	60.1
Women’s reports of whether counseled or not		
Yes	82	22.4
No	284	77.6
Knows source of LAPMs		
Yes	355	97.0
No	11	3.0
Mentioned sources LAPMs		
Hospital	324	88.6
Health Center	18	4.9
Clinic	14	3.8
Private Health facilities	10	2.7
Heard information of LAPMs		
Yes	361	98.6
No	5	1.4
Source of information of LAPMs		
Radio	170	46.5
Television	152	41.5
News Paper	18	4.9
Pamphlet	20	5.5
Health personnel	6	1.6
Discussed with husband on LAPMs		
Not at all	179	48.9
Once/twice	146	39.9
More often	41	11.2
Husband attitude to LAPMs		
Approves	153	41.8
Opposes	198	54.1
Don’t know	15	4.1
Main decider on using LAPMs		
Self-	111	30.3
Husband	20	5.5
Both decide together	235	64.2

Among current short acting contraceptive users, 127 (34.7 %) desired to use LAPMs of contraceptives. From those who desired to use LAPMs 95 (74.6 %) desired to use Implants. For more than half, 51 (53.7 %) of the non-users of Implants the reason for not using Implant was fear of complications. Fear of complications was not only the reason for not using Implant but also for IUCD’s and Tubal ligation. For those who desired to use LAPMs of contraception, but are not using the methods, their reasons for husband/partner opposition, previous method inconvenient and proven health problems (Table 4).

**Table 4:** Percentage of methods preferred & reasons for not using LAPMs among women in the reproductive age group (15-49) in Shashemene town, West Arsi Zone, Oromia Region, 2012

Characteristics	Number	Percent
Currently used Short Acting Method preferred n = 262		
Yes	135	51.5
No	127	48.5
What LAPM was wanted? n = 127		
IUD	27	21.3
Implants	95	74.8
Tubal ligation	5	3.9
Reason for not using IUD n = 27		
Previously used methods inconvenient	4	14.8
Proven health problem	2	7.4
Fear of complication	15	55.6
Partner/husband opposed	6	22.2
Reason for not using Implant = 95		
Previously used methods inconvenient	10	10.5
proven health problem	4	4.2
Fear of complications	51	53.7
Partner/husband opposed	30	31.6
Reason for not tubal ligation n = 5		
Proven health problem	1	20.0
Fear of complications	3	60.0
Partner/husband opposed	1	20.0

Education of women was found to be an associated factors of unmet need of LAPMs of contraception, respondents who had less than secondary levels of education were found about four times more likely to have had unmet need of LAPMs of contraception than those respondents who had secondary and above levels of education AOR [3.8, 95 % CI: 2.9, 7.6; P < 0.001]. Women’s discussion with their partners was found to be an associated factor of unmet need of LAPMs of contraception, respondents who don’t discuss with their partners were found about three times more likely to have had unmet need of LAPMs of contraception than those respondents who discuss with their partner at least one times and more AOR [2.9, 95 % CI: 1.8, 9.6; P = 0.01]. Furthermore women’s reports of whether they were counseled or not to use LAPMs of contraceptives was found to be an associated factors of unmet need of LAPMs of contraception, respondents who were not properly counseled by health professionals to use LAPMs of contraception were found to be more than five times more likely to have had unmet need of LAPMs of contraception than those respondents who were properly counseled by health professionals to use LAPMs of contraception AOR [5.3, 95 % CI: 1.7, 11.2; P = 0.04]. The other important association found was women’s occupation, respondents who were categorized as housewives  were found to be about five times more likely to have had unmet need of LAPMs of contraception than those respondents who had other occupation AOR [4.7, 95 % CI:3.1, 11.3; P = 0.02]. In logistic regression model, age, parity, residence, religion, knowledge of places, monthly income and the number of children were not associated with unmet need of LAMPs of contraception (Table 5).

**Table 5:** Association of unmet need for LAPMs and its correlates among women in the reproductive age group (15-49) using contraception in health facilities in Shashemene town, West Arsi Zone, Oromia Region, 2012 (Multivariate table)

Determinants	Unmet need of LAPMs	Crude	Adjusted^*^
	Yes (%)	OR(95 % CI)	OR(95 % CI)
Educational status			
Illiterate/Primary (1-8)	82(42.1)	4.10(1.64, 8.11)	3.80(2.90,7.60)
Secondary (9-10) & above	40(23.4)	1.00	1.00
Number of pregnancy			
Zero	10(35.7)	1.00	1.00
One/two	46(33.1)	1.56(0.54,3.35)	1.42(0.24,5.94)
Three/four	27(27.8)	1.16(0.48,4.69)	3.70(0.41,8.14)
Five or more	39(38.2)	1.74(1.86,7.88)	1.50(0.34,5.80)
Discussed with husband on LAPMs			
Not at all	69(38.1)	3.77(2.51,7.73)	2.90(1.80,9.60)
Once/twice	39(27.3)	3.79(0.85,8.17)	2.45(0.62, 5.43)
More often	14(33.3)	1.00	1.00
Main decider on using LAPMs			
Self/Husband	51(38.3)	1.35(1.62,6.33)	0.34(0.26,5.47)
Both decide together	71(30.5)	1.00	1.00
Women’s reports counseled or not			
Yes	26(29.9)	1.00	1.00
No	96(34.4)	5.60(2.44,12.67)	5.30(1.70,11.20)
Number of living children			
Zero	18(29.5)	1.00	1.00
One/two	42(31.8)	0.32(0.58,4.55)	0.66(0.38,6.71)
Three/four	29(32.2)	3.81(0.43,6.41)	2.84(0.63,5.42)
Five or more	33(39.8)	3.11(2.75,8.19)	4.16(0.54,8.11)
Occupational status			
House wife	54(43.5)	6.30(2.20,15.10)	4.70(3.10,11.30)
Government employee	19(27.5)	3.18 (0.16,1.36)	3.70(0.31,3.09)
Daily laborer	10(47.6)	1.15(0.56,4.07)	1.55(0.65,3.11)
Student	17(34.7)	3.11(0.26,5.35)	1.19(0.12,3.35)
Merchant	22(21.4)	1.00	1.00
Husband attitude to LAPMs			
Approves	44(22.7)	0.29(0.45,3.74)	1.36(0.28,7.78)
Opposes	73(46.5)	2.17(2.94,10.33)	0.16(0.33,25.33)
Don’t know	5(33.3)	1.00	1.00

^*^Adjusted for: Number of pregnancy, Main decider on using LAPMs, Number of living children and Husband attitude to LAPMs

## Discussion

In this study the women education was found to be one of associated factors of unmet need of LAPMs of contraception. This finding is similar to a study done in Ethiopia and Sri Lanka [17, 18, 19]. So, effort should be on empowering every woman for education as stated in Millennium Development Goal 2.

Women’s discussion with their partners was also found to be an associated factor of unmet need of LAPMs of contraception, respondents who don’t discuss with their partners were found about four times more likely to have had unmet need of LAPMs of contraception than those respondents who discuss with their partner at least ones or more. This is similar to studies done in Jimma and Uganda [20, 21]. This tells us the importance of partner discussion and support on family planning.

A study done in Jimma (Ethiopia) shows women’s being counseled by health professionals was one of associated factors for unmet need for LAPMs [20]. This study also reveals the same result that women who were not properly counseled by health professionals to use LAPMs of contraception were found more than six times more likely to have had unmet need of LAPMs of contraception than those women with proper consultation. This implies that FP service providers should recognize their big responsibility and accountability on providing women who were using modern FP with appropriate counseling about LAPMs.

The other important association with unmet need of LAPMs was the woman’s occupation, respondents who were categorized as housewife were found about six times more likely to have had unmet need of LAPMs of contraception than those respondents with other occupation similar to other studies done in developing countries [22]. Substantial increase in the use of maternal health services can be achieved by accelerating socioeconomic development and effectively addressing basic human needs of schooling, economic welfare, and gender-based discrimination [22].

The study suffers from the usual limitation of cross sectional study. The study could not discover any information from the service providing side of LAPMs. Furthermore, this study was not triangulated by qualitative study. Since the study was institutional based, that might undermine generalizing the result to the general population, and only female contraceptive users were selected, so it was not comprehensive enough to represent all males and females who are desire to use but not using LAPMs.

## Conclusion

Unmet need of LAPMs of contraception in health facilities in Shashemene town was high. Factors significantly associated with unmet need of LAMPs of contraception were women’s education, spouse discussion and women counseling. Hence we recommend policy makers and different stakeholders to focus on these major factors associated with LAPMs to increase the service.

## Abbreviations

AOR: Adjusted Odds Ratio
CPR: Contraceptive Prevalence Rate
E.C.: Ethiopian Calendar
EDHS: Ethiopian Demographic Health Survey
ETB: Ethiopian Birr
FP: Family Planning
IUCD: Intra Uterine Contraceptive Device
LAPMs: Long Acting and Permanent Methods
NGO: Non Governmental Organization
RH: Reproductive Health
